# How to Write a Medical Paper

**Published:** 1897-04

**Authors:** 


### How to Write a Medical Paper.

Dr. W. P. Northrup, of New York, makes the following excellent suggestions: "Scratch out your introduction and begin where the subject really begins. Condense the body of the paper. End the paper where the subject ends. Successful papers, almost without exception, are those written with one definite and predominating thought, on which every fact is brought to bear, and toward which every argument is directed. Conclusions alone are, as a rule, sufficient, with pertinent facts so marshaled as to give them proper support. The various minute details of the stages by which these conclusions are reached are usually uninteresting and had better be touched on lightly or omitted entirely. An expert editor by remorselessly stripping away the padding, is usually able to make an abstract that will present all the author's ideas and conclusions in one-fourth the space of the original paper. Many a man who has had something of real value to say has first smothered the life out of it with padding, and then dug a grave and buried it in the midst of a five-column paper compiled from some text-book. It would be far better for professional literature if every man would content himself with writing what he really knows, instead of writing what he has only read. One new fact discovered, one new, live, practical idea, is a sufficient subject for one paper, though it may be a short one. Two or three subjects for a single paper will render it weak or actually inert. A shotgun is adapted to small game, but large game is only brought down with a rifle. A single paper on a live subject, if it hits the mark squarely, will do more to establish a man's reputation than ten diluted and watery compilations."—*Western Med. Review.*

